# The Outcome of a Single-Incision Sling versus Trans-Obturator Sling in Overweight and Obese Women with Stress Urinary Incontinence at 3-Year Follow-Up

**DOI:** 10.3390/jcm8081099

**Published:** 2019-07-25

**Authors:** Hui-Hsuan Lau, Sugarmaa Enkhtaivan, Tsung-Hsien Su, Wen-Chu Huang

**Affiliations:** 1 Department of Medicine, Mackay Medical College, New Taipei City 252, Taiwan; 2 Division of Urogynecology, Department of Obstetrics and Gynecology, Mackay Memorial Hospital, Taipei 104, Taiwan; 3 Mackay Medicine, Nursing and Management College, New Taipei City 251, Taiwan; 4 Department of Obstetrics and Gynecology, Hsinchu Mackay Memorial Hospital, Hsinchu 300, Taiwan; 5 ASE Gynecological Surgery Hospital, Ulaanbaatar 16065, Mongolia

**Keywords:** body mass index, single incision sling, stress urinary incontinence, trans-obturator mid-urethral sling

## Abstract

*Background*: Being overweight or obese is a risk factor for incontinence and has negative impacts on the surgical outcomes. Compared with trans-obturator sling (TOS), single incision sling (SIS) is a new generation of anti-incontinence surgery. However, the data on SIS in overweight and obese women remains limited. *Methods*: This retrospective study analyzed the objective and subjective cure rate of overweight and obese women who underwent sling surgeries. Other evaluations included valid questionnaires to assess quality of life and 1-hour pad test to quantify urine leakage. Surgical characteristics and adverse events were also analyzed. *Results*: A total of 217 patients were analyzed with a median follow-up period of 37.3 months (range, 9–84 months). For overweight and obese patients, the objective and subjective cure rate were comparable (all *p* > 0.05). However, the SIS group had worse post-operative incontinence-related symptom distress (*p* < 0.001) and 1-hour pad test (*p* = 0.047). On the other hand, SIS had a shorter surgery time (*p* = 0.017) and lower pain score (*p* < 0.001). *Conclusions*: Compared with TOS, SIS had non-significant cure rates in the overweight and obese women. SIS had worse urine leakage and incontinence symptoms, but less surgical and wound pain in obese women. Thorough pre-operative counseling is necessary.

## 1. Introduction

Stress urinary incontinence (SUI) is defined by the International Urogynecological Association and International Continence Society as involuntary loss of urine on effort, physical exertion, sneezing, or coughing [1]. Numerous risk factors are associated with SUI including aging, obesity, pregnancy, childbirth, hysterectomy, menopause, or heavy lifting work [2]. Of these factors, obesity has been reported to be highly associated with SUI independently of other risk factors in several studies [3,4]. The World Health Organization defines a body mass index (BMI) of 25.0–30.0 kg/m^2^ as overweight and >30 kg/m^2^ as obesity [5]. A urodynamic evaluation study reported an association between increased BMI and increased intra-abdominal pressure [6], and another cohort study reported a dose-response effect of body weight on SUI symptoms, with each 5-unit increase in BMI being associated with a 20–70% increase in the risk of incontinence [4]. Although the relationship between obesity and SUI has been well documented, relatively few studies have investigated the safety and efficacy of surgical treatments among overweight and obese women. A mid-urethral sling is currently the most effective treatment for incontinence [7]. The first mid-urethral sling, tension-free vaginal tape, was developed by Ulmsten in 1996 with promising long-term results [8]. Subsequently, the trans-obturator sling (TOS) was launched in 2001 to reduce the risk of complications and was reported to have similar efficacy [8]. In 2006, the single-incision sling (SIS), a less invasive procedure, was developed as an alternative procedure to reduce inguinal pain after TOS [7].

Obese women may have more muscle mass or subcutaneous tissue, which makes the TOS needle passing through the obturator foramen difficult and increases the risk of complication. SIS has a shorter tape and a self-fixation anchoring system which makes it easy to be inserted into the obturator internus muscles; however, its durability remains unclear. Obesity may lead to chronic stress on the pelvic floor and on the mid-urethral sling, which may result in surgical failure over time. As a result, to assess the efficacy and safety of mid-urethral sling used in obese or overweight women is important. There are a few studies investigating the impact of obesity on outcomes of anti-incontinence surgery. In these studies, TOS is an effective and safe procedure to treat SUI, with minimal complications [9,10]. However, the success rates appeared to be lower in the women with obesity compared to those without obesity, even though the difference was not significant [9,10]. To date, few studies have reported on the outcomes of the SIS in women with obesity or overweight, and the results have been conflicting [11,12]. One study showed that the SIS seems to be an effective treatment irrespective of BMI [11], while another reported worse outcomes in women with obesity after SIS [12]. The aim of this study was to compare the subjective and objective cure rates of SIS versus TOS in overweight and obese women. The postoperative quality of life and surgical complications were also reported.

## 2. Experimental Section

We reviewed the records of women who received the SIS or TOS in a tertiary medical center between January 2010 and December 2016. Evaluations included a detailed personal history, pelvic examination with cough test, urine analysis, and 1-h pad test. Urodynamic measurements and quality of life assessments were performed pre- and postoperatively. We excluded the patients with an active urinary or vaginal infection at the time of surgery, recurrent SUI, concomitant pelvic surgery, mixed incontinence, and those who did not complete all investigations. The patients were stratified into two groups: those with a BMI 25.0–30.0 kg/m^2^ as the overweight group, and those with a BMI >30 kg/m^2^ as the obese group. This study was approved by the Institutional Review Board (Mckay Memorial Hospital, MMH-I-S-578, 19 June 2008) of our hospital.

Complete multichannel urodynamic studies including free uroflowmetry, filling and voiding cystometry, and urethral pressure profile were performed in each patient. All data were recorded and analyzed using a Medical Measurement Systems (MMS UD-200, Enschede, The Netherlands). quality of life was assessed using the short forms of the Urogenital Distress Inventory (UDI-6) and Incontinence Impact Questionnaire (IIQ-7) for incontinence-related symptoms and quality of life, with a higher score indicating worse symptoms and poorer quality of life [13]. The short form of the Pelvic Organ Prolapse/Urinary Incontinence Sexual Function Questionnaire (PISQ-12) was used to evaluate sexual function in women with pelvic floor dysfunction, with a higher score indicating a better quality of life [14]. Postoperative follow-up was scheduled at 1 week after discharge, then at 1, 3, 6, and 12 months, and yearly thereafter.

The SISs used included Mini-Arc^®^ (American Medical Systems, Minnetonka, MN, USA) and Solyx^®^ (Boston Scientific Corp; Natick, MA, USA) slings. The only TOS used was the TVT-O^®^ sling (Ethicon Inc., Johnson & Johnson, Somerville, NJ, USA). The objective success rate was defined as no urine leakage during the stress test in the filling phase of urodynamic studies. Subjective cure was defined as no subjective leakage after surgery. Minor leakage and satisfaction with the procedure were considered improvements. Worse leakage or dissatisfaction was defined as subjective failure. The subjective success rate was defined as the sum of the subjective cure and improvement rates. Surgical characteristics and adverse events were also reviewed, including operation time, blood loss, bladder perforation, inguinal pain, mesh erosion, urinary retention, and postoperative urinary tract infections. Statistical analysis was performed using the independent t-test or Mann-Whitney U test for continuous variables, and the chi-square or Fisher’s exact test for categorical variables. A *p* value of <0.05 was considered to be statistically significant. Statistical analysis was performed using the Statistical Package for the Social Sciences version 20 (SPSS Inc., IBM Corp., Armonk, NY, USA).

## 3. Results

A total of 723 women received a mid-urethral sling for SUI during the study period, of whom 384 were overweight or obese. After excluding 43 concomitant pelvic reconstruction surgeries, 29 cases of recurrent SUI, 36 cases of mixed incontinence, and 59 cases with missing data, 217 patients were eligible for the analysis. The median follow-up duration was 37.3 months (range, 9–84 months). All of the surgical procedures were performed by two experienced surgeons. Urodynamic measurements and quality of life evaluations were conducted by three technicians. 

The baseline characteristics of the women who underwent SIS and TOS are shown in Table 1. There were no significant differences between the two groups in terms of mean age, BMI, parity, menopause, intrinsic sphincter deficiency, weight of 1-hour pad test, incontinence-related quality of life, symptom distress, and sexual quality of life (all *p* > 0.05). Table 2 demonstrates the surgical outcomes in the overweight women after SIS or TOS. There were no significant differences regarding the subjective and objective cure rates, de novo symptoms, and postoperative quality of life. 

For the women with obesity, there were no differences in the objective and subjective cure rates after SIS or TOS; however, incontinence-related symptom distress (6.7 ± 2.0 vs. 3.0 ± 3.3, *p* < 0.001) and post-operative 1-h pad test (9.2 ± 27.5 g vs. 3.6 ± 16.5 g, *p* = 0.047) were worse in the SIS group (Table 3). Surgical characteristics and adverse events in the two groups are shown in Table 4. The operation time (7.9 ± 3.7 min vs. 9.1 ± 3.5 min, *p* = 0.017) and postoperative pain score (0.5 ± 0.9 vs. 1.5 ± 2.1, *p* < 0.001) were better in the SIS group. Neither of two groups had long-term adverse effects, nor mesh extrusion. Regarding the effect of BMI on SIS or TOS, the women with obesity in both groups had a lower success rate, particularly in the SIS group, which almost reached statistical significance (objective and subjective cure, *p* = 0.068 and 0.058, respectively). In addition, obese women had significant worse 1-h pad test than overweight women in the SIS group (9.2 ± 27.5 g vs. 3.0 ± 18.4 g, *p* < 0.001) (Table 5).

## 4. Discussion

In this study, we showed that the overweight or obese women who received either the SIS or TOS reported an improvement in their incontinence symptoms and quality of life as shown by the significant decrease in the 1-h pad test, and the mean scores on the UDI-6 and IIQ-7 questionnaires. Nevertheless, compared to the overweight women, those with obesity in the SIS group had a worse 1-h pad test and incontinence symptom distress than those in the TOS group. Furthermore, the cure rate in the women with obesity tended to be lower at a median of 37.3 months follow-up, particularly in the SIS group.

With regards to the cure rate of anti-incontinence surgery in patients with a high BMI, Brennand et al. reported an 86% success rate for TOS in women without obesity (BMI <30 kg/m^2^) compared to 68% in those with obesity (BMI >30 kg/m^2^) [9]. Another study reported TOS success rates of 87%, 79%, and 72% in women with normal weight (BMI <23 kg/m^2^), overweight (BMI 23–27.7 kg/m^2^), and obesity (BMI >27.5 kg/m^2^), respectively, with no significant differences after 2 years of follow-up [10]. Both of these studies demonstrated that the patients had an improved quality of life and incontinence-related symptoms regardless of their BMI. SIS is a third-generation mid-urethral sling, and it was developed with the concept of using a shorter tape through a single vaginal incision to avoid the possible complications associated with the retro-pubic route and groin pain with the trans-obturator route. Very few studies have reported the surgical outcomes of SIS in overweight and obese patients, although obesity is considered to be an important risk factor for the failure of anti-incontinence surgery [4]. To the best of our knowledge, only four studies have discussed this issue. Meschia et al. conducted a retrospective, single-arm study with 1 year of follow-up, and reported success rates of 91.3% in women with a BMI <25 kg/m^2^, 83.5% in those with a BMI 25–30 kg/m^2^, and 75% in those with a BMI >30 kg/m^2^ [12]. A significant higher failure rate was noted in the women with obesity (OR 3.74; 95% CI 1.19–11.76) who underwent SIS surgery. Frigerio et al. conducted a retrospective, single-arm study with a mean follow-up period of 23.2 months and reported a cure rate of 87.6% for women with normal weight, 86.8% for those with overweight, and 81.5% for those with obesity [11]. The objective and subjective cure rates were promising in both the obese and non-obese groups without significant differences. Morán et al. conducted a prospective, single-arm study with a mean follow-up period of 22.3 months and reported objective and subjective cures of 83% and 88% irrespective of the BMI [7]. Oliveira et al. conducted a prospective, single-arm study with a mean follow-up period of 12 months and reported postoperative improvement rates in of 94% and 90% in women with and without obesity, respectively [15]. Although these single-arm studies showed conflicting outcomes, our data are similar to those reported by Meschia et al. in that the objective and subjective cure rates of SIS were 76% and 79%, respectively, with a median follow-up period of 37.3 months in the women with obesity. In addition, the obese group also had worse postoperative incontinence-related symptoms and pad urine leakage. The SIS was recently investigated in a cohort of women with an older age and overweight in Brazil, and the results showed that the success rate was better in those with a lower BMI [16]. The authors used a BMI cut-off value of 30 kg/m^2^ and reported that the patients’ satisfaction and pad use were significantly worse in the obese group (BMI >30 kg/m^2^), which is similar with our results.

Nambiar et al. conducted a Cochrane Database Review in 2017 and showed that the SIS was associated with significantly lower postoperative pain and shorter operation time compared to either a retropubic or transobturator mid-urethral sling [17]. Our data demonstrated a similar complication rate in both groups; however, SIS had advantages regarding less pain and shorter surgical time. In Nambiar et al.’s systemic review, the authors concluded that there was not enough evidence to draw a conclusion on the subjective and objective outcomes with regards to SIS versus other slings [17]. However, they emphasized that the fixation mechanism was important. With the SIS, differences in fixation mechanisms may influence the outcomes or even the main reasons for failure. The shorter tape is a strong point, however, it may also be a weak point, as the lack of robustness of the sling tip anchored to the obturator membrane can cause a problem with the fixation. Moreover, obesity plays a role in pushing the pelvic floor downwards thereby reducing the durability of the sling and worsening the continence mechanism. Another reason may be due to difficulty in the insertion-fixation mechanisms. Unlike TOS, in which the tape is passed through a skin or vaginal incision, the self-fixating tip of the SIS is difficult to visualize and adjust the tension. Our study showed that the overweight and obese women who received either the SIS or TOS had an improved quality of life. Nevertheless, despite the comparable cure rate, the effect of BMI in the SIS group almost reached statistical significance. As a result, we suggest that thorough counselling is mandatory for women with obesity who choose SIS as treatment for SUI.

This study has inherent limitations due to its retrospective design. Another limitation is the absence of a control-group comprising women who are not overweight or obese. However, the strengths include that all participants completed pre-and postoperative questionnaires. In addition, women who received a TOS, which is recognized as an effective treatment for SUI, served as a comparison group. Another strength is that we had a relatively longer follow-up period. Since overweight and obesity are associated with chronic increased intra-abdominal pressure, which is a risk factor for stress incontinence, a longer-term follow-up is beneficial.

## 5. Conclusions

Although SIS had a similar cure rate for the overweight and obesity women at a mean of 37.3 months follow-up, obesity had a more negative impact on shorter sling tape surgery. Thorough preoperative counseling is necessary for women with obesity considering the SIS as treatment for SUI.

## Figures and Tables

**Table 1:** Baseline characteristics of the women who received a single-incision or trans-obturator sling.

	Single-Incision Sling (*n* = 106)	Trans-Obturator Sling (*n* = 111)	*p*
Age (years)	55.9 ± 11.1	54.3 ± 9.0	0.421
Parity (*n*)	3.1 ± 1.3	3.3 ± 1.6	0.552
Body mass index (kg/m^2^)	28.9 ± 5.6	29.5 ± 6.1	0.857
Postmenopausal status (*n*)	42 (43%)	37 (41%)	0.318
Previous prolapse repair surgery (*n*)	21 (20%)	31 (29%)	0.113
Intrinsic sphincter deficiency (*n*)	9 (8%)	12 (11%)	0.365
Hormonal therapy (*n*)	6 (6%)	8 (9%)	0.521
1-hour pad test (g)	49.8 ± 49.5	40.9 ± 29.3	0.329
Quality of life			
UDI-6	29.8 ± 17.6	31.4 ± 18.9	0.377
IIQ-7	25.9 ± 16.4	27.7 ± 20.8	0.238
PISQ-12	34.5 ± 16.2	33.6 ± 15.6	0.711

Data are presented as mean ± standard deviation or as number (percent) of patients. BMI: Body mass index, UDI-6: The short form of Urogenital Distress Inventory, IIQ-7: The short form of Incontinence Impact Questionnaire, PISQ-12: The short form of the Pelvic Organ Prolapse/Urinary Incontinence Sexual Function Questionnaire.

**Table 2:** Outcomes of the overweight women (BMI: 25–29.9 kg/m^2^) after receiving a single-incision sling or trans-obturator sling.

	Single-Incision Sling (*n* = 72)	Trans-Obturator Sling (*n* = 71)	*p*
Objective cure (*n*)	64 (89%)	62 (87%)	0.488
Subjective cure (*n*)	66 (92%)	64 (91%)	0.489
De novo urgency (*n*)	6 (8%)	8 (11%)	0.272
De novo voiding difficulty (*n*)	3 (4%)	5 (7%)	0.352
1-hour pad test (g)	3.0 ± 18.4	3.3 ± 15.1	0.361
Quality of life			
UDI-6	2.7 ± 2.1	3.2 ± 3.3	0.473
IIQ-7	2.1 ± 3.9	3.3 ± 4.8	0.257
PISQ-12	37.8 ± 19.1	36.2 ± 16.3	0.896

Data are presented as mean ± standard deviation or as number (percent) of patients.

**Table 3:** Outcomes of the women with obesity (BMI ≥ 30 kg/m^2^) after receiving a single-incision sling or trans-obturator sling.

	Single-Incision Sling (*n* = 34)	Trans-Obturator Sling (*n* = 40)	*p*
Objective cure (*n*)	26 (76%)	32 (80%)	0.465
Subjective cure (*n*)	27 (79%)	34 (85%)	0.372
De novo urgency (*n*)	3 (9%)	3 (8%)	0.582
De novo voiding difficulty (*n*)	1 (3%)	3 (8%)	0.371
1-hour pad test (g)	9.2 ± 27.5	3.6 ± 16.5	0.047
Quality of life			
UDI-6	6.7 ± 2.0	3.0 ± 3.3	<0.001
IIQ-7	6.1 ± 7.9	6.2 ± 5.9	0.573
PISQ-12	30.7 ± 17.1	29.1 ± 16.0	0.479

Data are presented as mean ± standard deviation or as number (percent) of patients.

**Table 4:** Surgical characteristics and adverse events for the women receiving a single-incision sling or trans-obturator sling.

	Single-Incision Sling (*n* = 106)	Trans-Obturator Sling (*n* = 111)	*p*
Operation time (min)	7.9 ± 3.7	9.1 ± 3.5	0.017
Blood loss (mL)	5.8 ± 3.3	6.2 ± 3.4	0.343
Hospital stay (day)	1.5 ± 1.0	1.7 ± 0.5	0.458
Bladder perforation (*n*)	3 (3%)	5 (5%)	0.387
Pain (VAS score)	0.5 ± 0.9	1.5 ± 2.1	<0.001
Urinary tract infection (*n*)	6 (6%)	5 (5%)	0.468
Wound hematoma (*n*)	0 (0%)	3 (3%)	0.132
Temporal urine retention treated with catheter (*n*) *	3 (3%)	5 (4%)	0.526

* Women with two consecutive spontaneous voids of ≥200 mL with measured residual urine volumes of >100 mL within 1 week after sling surgery.

**Table 5:** Objective and subjective results after receiving a single-incision sling or trans-obturator sling.

**Single-Incision Sling**	**Overweight Patients (*n* = 72)**	**Obese Patients (*n* = 34)**	***p***
Objective cure (*n*)	64 (89%)	26 (76%)	0.068
Subjective cure (*n*)	66 (92%)	27 (79%)	0.058
1-hour pad test (g)	3.0 ± 18.4	9.2 ± 27.5	<0.001
**Trans-Obturator Sling**	**Overweight Patients (*n* = 71)**	**Obese Patients (*n* = 40)**	***p***
Objective cure (*n*)	62 (87%)	32 (80%)	0.250
Subjective cure (*n*)	64 (91%)	34 (85%)	0.175
1-hour pad test (g)	3.3 ± 15.1	3.6 ± 16.5	0.493
